# The more pieces, the better the puzzle: sperm concentration increases gametic compatibility

**DOI:** 10.1002/ece3.1684

**Published:** 2015-09-17

**Authors:** Craig D. H. Sherman, Emi S. Ab Rahim, Mats Olsson, Vincent Careau

**Affiliations:** ^1^ Centre for Integrative EcologySchool of Life and Environmental SciencesDeakin UniversityWaurn PondsVictoria3216Australia; ^2^ School of Biological SciencesUniversiti Sains Malaysia11800MindenPenangMalaysia; ^3^ School of Biological SciencesThe University of SydneySydneyNew South Wales2006Australia

**Keywords:** Cryptic female choice, genotype by environment, good genes, *Mytilus*, polyandry, quantitative genetics, sperm competition

## Abstract

The genetic benefits individuals receive from mate choice have been the focus of numerous studies, with several showing support for both intrinsic genetic benefits and compatibility effects on fertilization success and offspring viability. However, the robustness of these effects have rarely been tested across an ecologically relevant environmental gradient. In particular, sperm environment is a crucial factor determining fertilization success in many species, especially those with external fertilization. Here, we test the importance of sperm environment in mediating compatibility‐based selection on fertilization using a factorial breeding design. We detected a significant intrinsic male effect on fertilization success at only one of four sperm concentrations. Compatibility effects were significant at the two highest sperm concentrations and, interestingly, the magnitude of the compatibility effect consistently increased with sperm concentration. This suggests that females are able to modify the probability of sperm–egg fusion as the amount of sperm available increases.

## Introduction

It is widely accepted that mate choice occurs across a diverse range of taxa; however, the benefits females receive from such choice are still the focus of considerable debate (Andersson 1994; Colegrave et al. 2002; Kokko et al. 2003; Mays and Hill 2004; Andersson and Simmons 2006). In theory, females can obtain direct benefits and/or indirect genetic benefits from being choosey (Andersson 1994; Kokko et al. 2003). In mating systems where males provide direct benefits to females (e.g., nutritional resources, parental care, protection, shelter), females will often choose males that provide the most beneficial resources for them or their offspring. Yet females can still be highly selective, even when males provide no resources other than sperm for fertilization (i.e., nonresource‐based mating systems). In the absence of any direct benefits, males may be chosen because they provide genetic benefits that will increase the fitness of a female's offspring (Tregenza and Wedell 2000; Hunt et al. 2004; Nordeide 2007). Thus, the evolution of mate choice for indirect genetic benefits requires there to be variation in the fitness of offspring produced by a female if she mated with different males.

Broadly speaking, genetic benefits can be divided into two main types: (1) good gene benefits that arise from intrinsic genetic variation in male fitness and (2) genetic compatibility effects that arise from the interaction between mating partners (Tregenza and Wedell 2000; Neff and Pitcher 2005; Puurtinen et al. 2009). Mate choice based on good genes suggests that females should choose males with a high breeding value for fitness to increase the genotypic fitness value of their offspring (Andersson 1994; Colegrave et al. 2002). In contrast, the genetic compatibility hypothesis highlights the importance of the interaction between parental genotypes manifested in the offspring (Zeh and Zeh 1996, 1997; Nordeide 2007; Thünken et al. 2012). Under this scenario, it is how well the genes from two parents function together that determines the offspring fitness (Tregenza and Wedell 2000; Neff and Pitcher 2005). Hence, one “good” male may not be so for all females (Palumbi 1994; Zeh and Zeh 1996, 1997; Dziminski et al. 2008; Sherman et al. 2008b). Good gene and genetic compatibility effects can formally be defined in terms of their quantitative‐genetic components, with good gene effects relating to the additive genetic variance in fitness, while genetic compatibility is defined as the nonadditive genetic variance component of fitness (Neff and Pitcher 2005; Puurtinen et al. 2005, 2009). Partitioning the relative importance of good gene versus genetic compatibility effects can be performed using cross‐classified quantitative‐genetic breeding designs where females and males in an experimental block are mated in every pairwise combination (Neff and Pitcher 2005).

Broadcast spawning species with external fertilization are emerging as key model systems for understanding postmating sexual selection (Levitan 1998; Crean and Marshall 2008; Fitzpatrick et al. 2012; Evans and Sherman 2013). Indeed, selection on postmating mechanisms of mate choice should be particularly strong in sessile broadcast spawners, as postmating mechanisms provide one of the only opportunities for sexual selection to operate within this group. The release of gametes into the water column by multiple individuals provides an arena for sperm competition and/or cryptic female choice, mediated by sperm–egg interactions. Broadcast spawners also typically release large numbers of eggs and sperm that can be easily collected for controlled laboratory breeding experiments. Cross‐classified breeding designs provide a powerful approach for quantifying the importance of cryptic female processes in determining fertilization success that are difficult to assess in internal fertilizing species. A number of recent studies have demonstrated the use of broadcast spawners for use in assessing the relative importance of good genes and genetic compatibility effects (Merilä et al. 2004; Evans and Marshall 2005; Marshall and Evans 2005, 2007; Pitcher and Neff 2006, 2007; Dziminski et al. 2008).

One potential limit of previous quantitative‐genetic studies assessing good gene and genetic compatibility effects has been the use of only a single sperm concentration when carrying out fertilization assays. Yet variation in gamete concentrations in the water column during spawning events is known to be highly variable among and within natural populations and has important consequences on the probability of reproductive success or failure (Levitan and Petersen 1995; Levitan 1998, 2012; Styan 1998; Babcock et al. 2000; Yund 2000; Franke et al. 2002; Marshall et al. 2002). For example, females in high‐density populations often experience an excess of sperm and a high risk of polyspermy (Styan 1998; Yund 2000; Franke et al. 2002; Levitan and Ferrell 2006; Levitan et al. 2007; Levitan 2012); by contrast, females in low‐density populations face the risk of sperm limitation and low fertilization rates (Levitan and Petersen 1995; Levitan 1998, 2004; Yund 2000). Thus, it is expected that selection should act on mechanisms that allow females to modify the probability of sperm–egg fusion in relation to sperm environment to maximize fertilization success. Indeed, evidence from sea urchins suggest that gamete recognition proteins play an important role in determining fertilization success of males and females in relation to adult population density and the risk of polyspermy (Levitan and Petersen 1995; Levitan 1998, 2002, 2004, 2012; Levitan and Ferrell 2006; Levitan et al. 2007). A study by Levitan and Ferrell (2006) showed an interaction between the genotype frequency of the egg–sperm binding protein (Bindin) and spawning density in natural populations of the sea urchin *Strongylocentrotus franciscanus*. Common genotypes were selected under sperm‐limited conditions (low population density), and rare genotypes were selected under conditions of intense sperm competition and sexual conflict (high population density). In this instance, it appears that polyspermy avoidance is the main factor driving the evolution of gametic recognition proteins and compatibility effects within populations and not postzygotic benefits of mate choice (Levitan and Ferrell 2006). Thus, the availability of sperm has important influences on estimates of compatibility and intrinsic male fitness when assessing fertilization success. To our knowledge, however, the relative importance of intrinsic parental effects and compatibility effects on fertilization has never been tested across different sperm concentrations within a quantitative‐genetic framework.

The broadcast spawning mussel, *Mytilus galloprovincialis,* provides an ideal model system to study intrinsic parental effects and compatibility effects on fertilization success. Until recently, work in the context of mate choice during fertilization has focused on incompatibilities between individuals from distant populations or between sister taxa (e.g., McCartney and Lessios 2002); however, there are a growing number of studies that have explored these effects on fertilization between individuals from the same population (Palumbi 1999; Kupriyanova and Havenhand 2002; Evans and Marshall 2005; Marshall and Evans 2005, 2007; Sherman et al. 2008a). Nevertheless, few studies have examined how incompatibility effects vary along environmental gradients within a quantitative‐genetic framework. Here, we use a cross‐classified breeding design to assess how the variance observed in fertilization success is partitioned into intrinsic parental effects and compatibility effects, and how these components of variance change with sperm concentration.

## Materials and Methods

### Collection and spawning of animals

We collected mussel broodstock from a population east of Kirk Point (38°2′50.69″S, 144°38′8.28″E) Port Phillip Bay during the May winter spawning season. Mussels were transported to the Victorian Marine Science Consortium research laboratories at Queenscliff and held in flow‐through tanks using 1 *μ*m filtered seawater at ambient temperature (16°C). All animals were cleaned of epiphytes and used for spawning on the day of collection using the approach of Pettersen et al. (2010). Individuals were placed on a spawning table in approximately 6 cm of seawater and spawning induced using thermal shock. This involved progressively increasing seawater temperature from 16 to 24°C to trigger the release of gametes. Males and females were identified at the time of gamete release, rinsed with filtered seawater, and isolated into individual spawning chambers (120 × 175 × 70 mm). Individuals were allowed to continue to spawn for up to 20 min. Eggs were rinsed through a 125‐*μ*m mesh, and sperm through a 30‐*μ*m mesh, to remove any debris released from the adult mussel during spawning. Gamete solutions were then made up to a final volume of 300 mL. The concentration of sperm for each male was determined from three replicate counts using an improved Neubauer haemocytometer and sperm standardized to 6 × 10^6^ sperm mL^−1^. A serial sperm dilution was then carried out to obtain stock concentrations of 6 × 10^6^, 6 × 10^5^, 6 × 10^4^, and 6 × 10^3^ sperm mL^−1^. Egg concentrations were assessed from three replicate counts using a Beckman multisizer^™^ 3 Coulter counter and standardized to 6000 eggs mL^−1^ (stock egg solution).

### Fertilization trials

We used a cross‐classified breeding design (Lynch and Walsh 1998) involving four males crossed with four females in every pairwise combination. For each male–female cross, three replicate fertilization assays were carried out across each of four different sperm concentrations (see final concentration below), giving a total of 192 fertilization assays per block. A total of three fertilization blocks were carried out using four different males and four different females in each block. We conducted fertilization assays in 100‐mL sterile containers with a total fertilization volume of 75 mL. This consisted of 25 mL of the stock egg solution, and 50 mL of sperm resulting in a final egg concentration of 2000 eggs mL^−1^ and sperm concentrations of 4 × 10^3^, 4 × 10^4^, 4 × 10^5^, and 4 × 10^6^ sperm mL^−1^. All fertilization assays within a block were conducted immediately after gamete standardization and within 1 min of each other. The maximum time from initial gamete collection to fertilization was 60 min and is well within the time frame of 6–11 h reported for gamete viability in *Mytilus* species (Sprung and Bayne 1984). Fertilization assays were left at room temperature for 3 h before fixing with 10% formalin. For each replicate, a random subsample of approximately 100 eggs were observed under an inverted microscope at 400 ×  magnification and fertilized eggs distinguished from unfertilized eggs by counting the number of cells that had undergone cell division and/or the presence of a fertilization envelope. We performed a total of 576 crosses (i.e., 3 blocks × 4 males × 4 females × 4 concentration × 3 replicates); however, three replicates from a single male × female cross at sperm concentration of 4 × 10^4^ sperm mL^−1^ were excluded from the final analysis due technical error in sperm concentration standardization.

### Measures of condition and genetic relatedness

As body condition and genetic relatedness are known to have important influences on fertilization success, we wanted to control for these potential confounding effects in our analysis. We collected tissue samples and recorded size measurements [shell length (mm), shell width (mm), whole mass (g), and flesh mass (g)] from each brood parent. For estimates of genetic relatedness, we extracted DNA using Qiagen DNeasy Blood and Tissue kits following the manufacturer's instructions. All individuals were amplified for six polymorphic microsatellite loci (MgU2, MGE005, MT203, Med733, My029, and My650) (Presa et al. 2002; Yu and Li 2007; Gardeström et al. 2008; Lallias et al. 2009). As only six microsatellite markers were used, we choose the simplest measure of genetic similarity, allele sharing. Genetic similarity among individuals was calculated as the number of alleles shared (*B*_xy_) using the R package “Demerelate” as described in Li and Horvitz (Li and Horvitz 1953).

### Statistical analysis

We analyzed fertilization success using Markov chain Monte Carlo generalized linear mixed models in R (package MCMCglmm, (Hadfield 2010)). With this Bayesian mixed model approach, we modeled the proportion of eggs fertilized, following a binomial distribution, and obtained both an estimate of the components of variance and an estimate of the interval of credibility. All models were run for 1.3 × 10^7^ iterations, with a thinning interval of 10,000 (i.e., only one iteration from every 10,000 in the Markov chain was used to estimate the posterior distribution of the parameters to reduce the occurrence of autocorrelation between successive iterations), and a burn‐in of 3 × 10^6^ (i.e., we discarded the first 3 × 10^6^ models of the simulation to avoid issues with autocorrelation).

We included all the data (*n* = 573) in a single model, but allowed heterogeneous variance components according to sperm concentration. We included in the model fixed effects of flesh mass, an index of body condition (residuals of flesh mass regressed against body length) fitted separately for females and males, and an effect of genetic relatedness (*B*_xy_; see above). We also included a fixed categorical variable coding for sperm concentration to account for differences in fertilization success across sperm concentrations. The random effects used in the model included block (*V*_block_), identity of sire (*V*_sire_), identity of dam (*V*_dam_), and an interaction between the identity of sire and dam (*V*_sire:dam_). Because we included all 3 replicate values in the model, the residual variance (*V*_e_) represents the variance between replicates within a given pair of sire and dam. We modeled heterogeneous variance components for *V*_sire_, *V*_dam_, and *V*_sire:dam_ according to each concentration. Hence, there were 14 variance components estimated in this model (i.e., *V*_sire_, *V*_dam_, and *V*_sire:dam_ estimated separately for sperm concentrations and a single *V*_block_ and *V*_e_).

A necessary step in Bayesian statistical analyses is to set priors before running the models. The term “prior” refers to the prior distribution of a parameter before the data are analyzed. The level of information of the prior can vary from noninformative to highly informative. When knowledge about the relationship between the variables in the model is low, it is best to run the model with different priors and to check whether these different priors provide different posterior distributions (Hadfield 2010). We therefore ran the models using inverse Wishart priors (equivalent to an inverse gamma distribution with shape = scale = 0.001; V = 1, nu = 0.002) and parameter expanded priors (V = 1, nu = 1, alpha.mu = 0, alpha.V = 1000). Although we present results from the model using parameter expanded priors, the conclusions did not qualitatively change according to prior specifications. We also ran the model using the frequentist approach (ASReml‐R) to make sure the *V*_sire_ *, V*_dam,_ and *V*_sire:dam_ estimates fell within the 95% HPD (highest posterior density) obtained in the MCMCglmm.

By contrast to Gaussian data, with binomial data, it is not recommendable to compare different models using likelihood ratio tests (for REML models) or the deviance information criteria (an index produced by MCMCglmm models that balances the fit of the model based on the number of parameters used in the model). Thus, we cannot formally test whether model fit was improved by allowing heterogeneous variance components across sperm concentrations. Instead, we inspected the 95% HPD intervals associated with each fixed and random effect to check whether they overlapped. A 95% HPD interval contains most of the posterior distribution and is analogous to a confidence interval in the frequentist approach; two overlapping 95% HPD intervals indicate that the effect does not differ significantly (Hadfield 2010). Note that, as the lower limit of a variance component is bound to zero, its lower 95% HPD can be extremely close to, but cannot overlap zero. Thus, inspection of the HPDs cannot be formally used to test whether a variance component is significantly greater than zero (Hadfield 2010). Still, the 95% credible intervals around the variance estimates provide a measure of the precision of the estimate and allowed us to test whether variance components (*V*_sire_, *V*_dam_, and *V*_sire:dam_) differed across sperm concentrations.

## Results

**Figure 1:**
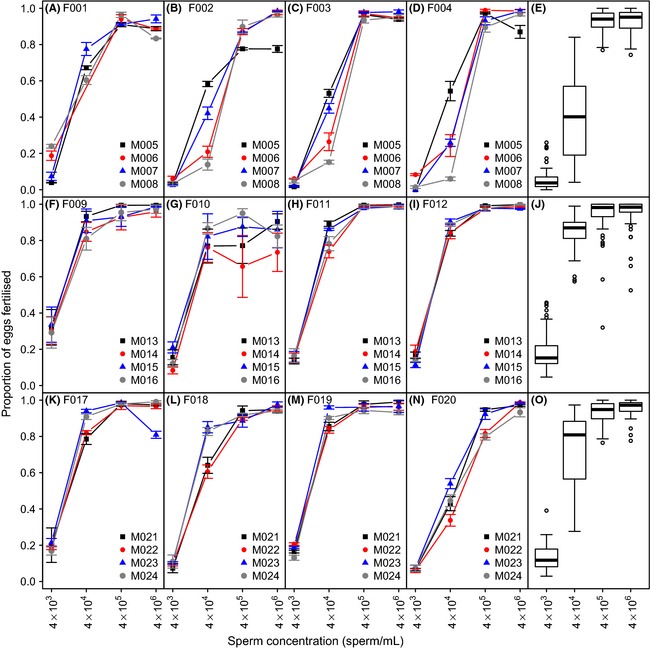
Proportion of eggs fertilized as a function of sperm concentration in 24 mussels (12 females and 12 males). For each experimental block, four females were paired with four males (and *vice versa*) at four different sperm concentrations (4 × 10^3^, 4 × 10^4^, 4 × 10^5^, and 4 × 10^6^ sperm mL^−1^). Each line within a panel represents a different male paired with that female. The rightmost panels show the pooled distribution of fertilization success for each of the 3 blocks.

Fertilization success increased with increasing sperm concentration; however, there was significant variation across males and females (Fig. 1). For example, at the second lowest concentration (4 × 10^4^ sperm mL^−1^), some males ranked consistently low or high across four females (e.g., males M008 and M005 in Fig. 1A–D). At the highest concentration (4 × 10^6^ sperm mL^−1^), however, the relative ranking of males was more variable across females (e.g., males M023 and M024 ranked differently depending on which females they were crossed with, see panels K‐N in Fig. 1). We also detected a decrease in fertilization success between some male–female combinations at the highest sperm concentration, suggesting variance in the degree of polyspermy between these combinations (e.g., panels A, D, G, and K, Fig. 1). We detected no effect of flesh mass or body condition on fertilization success in either sex (Table 1A). Pairs of males and females with higher allele sharing (*B*_xy_) tended to have lower fertilization success, but the effect of genetic relatedness was nonsignificant (*P *=* *0.096; Table 1A).

**Table 1:** Parameters from a mixed model of proportion of eggs fertilized across different sperm concentration in *Mytilus galloprovincialis*, fitted using a Bayesian approach. Shown are posterior modes and the 95% HPD (highest posterior density) intervals for (A) fixed effects of sperm concentration (as a categorical variable), female and male flesh mass and body condition, genetic relatedness (*B*_xy_), and (B) random effects of measurement block (*V*_block_), sire identity (*V*_sire_), dam identity (*V*_dam_), specific combinations of sires and dams (*V*_sire:dam_), and specific environment (*V*_e_; residual variance). *V*_sire_, *V*_dam_, and *V*_sire:dam_ were fitted heterogeneously for each sperm concentration

Level	Term	Posterior mode	95% HPD		*P*
			Lower	Upper	
(A) Fixed effects	Intercept	−0.33	−5.43	7.09	0.930
	Sperm concentration [4 × 10^4^]	3.24	2.22	3.92	<0.001
	Sperm concentration [4 × 10^5^]	5.25	4.53	6.19	<0.001
	Sperm concentration [4 × 10^6^]	5.89	4.90	6.51	<0.001
	Female flesh mass	−0.18	−0.55	0.10	0.166
	Male flesh mass	−0.06	−0.23	0.05	0.176
	Female body condition	−0.06	−0.59	0.41	0.890
	Male body condition	−0.03	−0.18	0.28	0.760
	*Genetic similarity (B*_xy_ *)*	−0.47	−1.20	0.11	0.096
(B) Random effects [sperm concentration]	*V*_block_	−0.53	<0.01	187	
	*V*_sire_ [4 × 10^3^]	0.11	<0.01	0.47	
	*V*_sire_ [4 × 10^4^]	0.47	0.14	1.54	
	*V*_sire_ [4 × 10^5^]	<0.01	<0.01	0.27	
	*V*_sire_ [4 × 10^6^]	0.01	<0.01	0.67	
	*V*_dam_ [4 × 10^3^]	0.34	0.11	1.17	
	*V*_dam_ [4 × 10^4^]	0.54	0.22	2.22	
	*V*_dam_ [4 × 10^5^]	0.60	0.23	2.41	
	*V*_dam_ [4 × 10^6^]	0.54	<0.01	2.00	
	*V*_sire:dam_ [4 × 10^3^]	<0.01	<0.01	0.07	
	*V*_sire:dam_ [4 × 10^4^]	<0.01	<0.01	0.12	
	*V*_sire:dam_ [4 × 10^5^]	0.16	0.03	0.40	
	*V*_sire:dam_ [4 × 10^6^]	0.37	0.15	0.94	
	*V*_e_	0.25	0.20	0.31	

**Figure 2:**
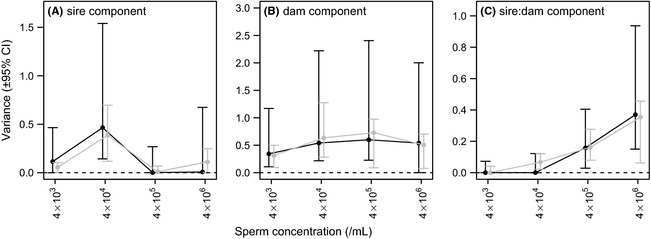
Variance in fertilization success attributed to (A) sire identity (*V*_sire_), (B) dam identity (*V*_dam_), and (C) the interaction between sire and dam identity (*V*_sire:dam_) across sperm concentrations (4 × 10^3^, 4 × 10^4^, 4 × 10^5^, and 4 × 10^6^ sperm mL^−1^) in mussels. Black lines show posterior modes and the 95% confidence intervals (CI; highest posterior density intervals) from the MCMCglmm model. Gray lines show estimates from the ASReml‐R model with 95% CI estimated using profile likelihoods. Estimates are significant if their lower CI does not overlap with 0 (dotted line).

Intrinsic male effects (*V*_sire_) accounted for a relatively low proportion of the variance in fertilization success (range: 0.00–0.47) and were not statistically greater than zero in three of four different sperm concentrations (Table 1B; Fig. 2A). The only sperm concentration at which the *V*_sire_ component was statistically greater than zero (i.e., 4 × 10^4^ sperm mL^−1^; Table 1B) is the concentration with the greatest variance in fertilization success (see Fig. 1E, J, and O). Maternal and common environmental effects (*V*_dam_) accounted for a relatively high proportion of the variance (range: 0.34–0.60) and were statistically greater than zero at three of the four sperm concentrations (Table 1B; Fig. 2B). The “sire×dam” interaction effects (*V*_sire:dam_) accounted for a relatively low proportion of the variance (range: 0.00–0.37) and were not statistically greater than zero in two of four different sperm concentrations (Table 1B; Fig. 2C). For example, the upper 95% credible interval at 4 × 10^3^ sperm mL^−1^ (<0.01 to 0.07) and 4 × 10^4^ sperm mL^−1^ (<0.01 to 0.12) are lower than the lower 95% interval at 4 × 106 (0.15–0.94), indicating significant differences in the interaction effect between males and females at different sperm concentrations (Table 1B). Most interestingly, the magnitude of the *V*_sire:dam_ component increased with sperm concentration (Table 1B; Fig. 2C).

## Discussion

Our analysis of fertilization success across an ecologically relevant range of sperm concentrations revealed that the sire and sire × dam effects varied greatly. The sire effect was significant at only one of four sperm concentrations (i.e., 4 × 10^4^ sperm mL^−1^), and the sire × dam interaction was significant at the two highest sperm concentrations (i.e., 4 × 10^5^ and 4 × 10^6^ sperm mL^−1^). Thus, had we conducted our experiment at a single concentration our conclusions on the relative importance of sire and compatibility effects would have depended on the sperm concentration used. The greater compatibility effect at higher sperm concentrations suggests that different male–female combinations may vary in their degree of polyspermy risk and that polyspermy avoidance, rather than postzygotic benefits of mate choice, may play an important role in driving sperm–egg compatibility in this system. This result is consistent with studies in the sea urchin *S*. *franciscanus* that have shown that the risk of polyspermy is an important driver of compatibility among males and females and is responsible for maintaining balanced polymorphism in both the egg and sperm recognition loci generating matched compatibility types (Levitan and Ferrell 2006; Levitan et al. 2007). Our results suggest that compatibility effects in particular need to be considered in context of the environment they are measured (see also Nystrand et al. 2011; Eads et al. 2012; Lymbery and Evans 2013).

Several studies of female choice have used a quantitative‐genetic framework to show various support for either good or compatible gene effects (Evans and Marshall 2005; Marshall and Evans 2005, 2007; Pitcher and Neff 2006, 2007; Ivy 2007; Bilde et al. 2008; Dziminski et al. 2008; Wedekind et al. 2008; Rodriguez‐Munoz and Tregenza 2009; Evans et al. 2010; Eads et al. 2012). However, the majority of these studies have been conducted under a standard homogenous environment, yet most organisms in nature are typically found across a range of environmental conditions. The relative importance of good and compatible gene effects (or compatibility driven by polyspermy risk) may not be consistent across these environments, with only a few studies to date having assessed the influence of the environment on the relative importance of good gene and genetic compatibility effects (Nystrand et al. 2011; Eads et al. 2012; Lymbery and Evans 2013). These studies have revealed complex interactions between male‐by‐female interaction components and the environments in which they are assessed. Thus, it is becoming increasingly clear that the assessment of the genetic benefits of mate choice should be considered within different environmental contexts. Our results are consistent with these studies and strongly indicate that compatibility effects on fertilization success change across sperm concentration, which can be highly variable in space and time in most broadcast spawning species.

Partitioning the relative importance of good gene versus genetic compatibility effects can be carried out using traditional quantitative‐genetic breeding designs, such as the cross‐classified breeding design that we used here (Lynch and Walsh 1998; Neff and Pitcher 2005). This design enables the variance observed in any number of traits expressed in the offspring to be partitioned into the underlying additive and nonadditive genetic variance. Arguably, fertilization rates are determined in part by sperm performance and egg quality, which is influenced by both genes and the environment of the parents (Snook 2005; Johnson et al. 2013). For example, ejaculate quality (size, sperm morphometrics, motility, and energetic capacity) have all been shown to be influenced by male condition or size (Evans and Geffen 1998; Skinner and Watt 2007; Burness et al. 2008). Thus, while the *V*_sire_ component that we quantified may contain additive genetic variance, it also contains some uncontrolled environmental variance component. Irrespective of the relative importance of additive genetic and environmental variances on our *V*_sire_ component, our result suggests that the greater the variation in offspring fitness in a population (in our case, fertilization success), the greater the opportunity for females to be choosey and potentially benefit from indirect genetic benefits. Indeed, the sire effect was significant only at 4 × 10^4^ sperm mL^−1^, which corresponds to concentration at which fertilization success was the most variable.

Our analysis of fertilization rates revealed that the *V*_sire:dam_ component increased with sperm concentration. Although it is relatively easy to imagine how the environment experienced by sires may have subsequently influenced their sperm performance and fertilization success across all females (see above), it is harder to understand why the environment experienced by a sire would predispose it to be more compatible with a certain female but not others. Thus, our results suggest that the importance of compatibility effects on fertilization increases with sperm concentration. Nevertheless, to rule out the possibility that environmental effects experienced by sires influenced compatibility, we suggest that future quantitative‐genetic experiments should use multigeneration pedigrees.

So what can explain the possible mechanism underlying the effect of sperm environment on compatibility effects based on the current knowledge of fertilization processes? Under conditions of sperm limitation, females face the risk of fertilization failure and should be less choosey about which sperm fertilize their eggs (Levitan and Petersen 1995; Yund 2000). However, as the amount of sperm available to an egg increases, females should become more “choosy” about which sperm fertilize their eggs. While the mechanisms underlying these cryptic sperm preferences remain unknown for the vast majority of broadcast spawners, it is becoming increasingly clear that egg–sperm interactions mediated via gamete recognition proteins are likely to play a crucial role (Vacquier 1998; Swanson and Vacquier 2002; Levitan 2012; Evans and Sherman 2013). These gamete recognition proteins are often involved in binding sperm to the egg membrane and facilitating the penetration of the sperm into the egg (Swanson and Vacquier 2002; Clark et al. 2006). More compatible sperm appear to be able to penetrate eggs more readily compared to less compatible sperm (Geyer and Palumbi 2003, 2005; Slaughter et al. 2008). As the number of sperm attaching to an egg increases, more compatible sperm should be at an advantage and penetrate the egg more readily compared to less compatible sperm. Thus, compatibility effects should become more pronounced with increasing sperm concentration (as seen in this study) or as the variation in compatible sperm attaching to an egg increases.

Another potential mechanism mediating compatibility effects across sperm concentrations is via chemoattractants. Recent studies in *M. galloprovincialis* have shown that sperm are capable of showing preference for particular females eggs based on the chemoattractants released by eggs and that this promotes assortative fertilizations between genetically compatible gametes (Palumbi 1994; Evans et al. 2012; Oliver and Evans 2014). However, if eggs can facultatively adjust the amount of chemoattractant released in relation to sperm concentration, then under low sperm concentration, eggs should release more chemoattractants to increase sperm attraction, but this may also allow less compatible sperm to find and fertilize the egg. In contrast, under high sperm concentrations, eggs should decrease the amount of chemoattractant released to minimize the potential for polyspermy. More compatible sperm may have an advantage if they are able to detect, locate, and fertilize the eggs quicker compared with less compatible sperm when chemoattractant concentrations are low.

While our results show an increase in compatibility effects with increasing sperm concentration, it should be noted that these represent noncompetitive fertilizations trials. We expect that under conditions of sperm competition, where the sperm from multiple males compete to fertilize eggs, compatibility effects may be even more pronounced (Sherman et al. 2008b, 2010). Future studies should conduct sperm competition trials at different sperm concentrations to test whether genetic compatibility also varies with sperm concentration under more natural conditions (i.e., more than one male spawning at the same time). Additionally, several quantitative‐genetic studies are required to determine how the relative importance of good genes and compatible genes benefits change across a range of other potentially relevant environmental gradients such as number of competing males, mating order, differential maternal effects, pH, and temperature (Nystrand et al. 2011; Eads et al. 2012; Lymbery and Evans 2013). Taken together, our results emphasize the importance of considering fertilization success across multiple ecologically relevant environments when considering the individual (and genetic) basis of mate choice.

## Data Archiving

All data used in the analyses to create the figures and tables are available via the Dryad Digital Repository: doi:10.5061/dryad.88v8j.

## Conflict of Interest

None declared.
